# eDNA reveals extraordinary fish diversity in the Urauchi River, Iriomote Island, Japan, a UNESCO World Heritage Site

**DOI:** 10.7717/peerj.21399

**Published:** 2026-06-24

**Authors:** Bernadeth Grace S. Pananganan, Marizka G. Juliano, Yukinobu Isowa, Maria Daniela Artigas Ramirez, Hiroyuki Motomura, Tadashi Kajita

**Affiliations:** 1 Iriomote Station, Tropical Biosphere Research Center, University of the Ryukyus, Taketomi, Okinawa, Japan; 2 United Graduate School of Agricultural Sciences, Kagoshima University, Kagoshima, Kagoshima, Japan; 3 Center of Applied Biotechnology, Faculty of Experimental Science and Technology, Department of Biology, University of Carabobo, Venezuela; 4 The Kagoshima University Museum, Kagoshima, Kagoshima, Japan

**Keywords:** Iriomote, Transitional zone, Megadiversity, Subtropical ecosystem, Ichthyofaunal diversity

## Abstract

Robust biodiversity monitoring is essential for the management of UNESCO World Natural Heritage Sites; yet, systematic assessments of ichthyofaunal communities remain limited. Here, we apply environmental DNA (eDNA) metabarcoding to surface water samples collected in the Urauchi River (Iriomote Island, Japan), a United Nations Educational, Scientific and Cultural Organization (UNESCO) World Heritage site and one of Japan’s most fish-diverse rivers. Surface water samples were collected at 10–11 sites along the 18.8 km stretch in May 2021 and November 2024. Using MiFish primers, high-throughput Illumina sequencing, and QIIME 2 pipeline, we obtained 35.7 million quality-filtered reads, from which 332 operational taxonomic units (OTUs) belonging to Actinopterygii and Chondrichthyes were assigned to 254 species, 64 genera, and 11 families, along with three unannotated OTUs due to low taxonomic resolution. Species richness peaked in the mid-to-lower estuary within dense mangrove zones, with α-diversity indices (Shannon, Simpson) increasing downstream. β-Diversity analyses [Bray–Curtis, Non-metric Multidimensional Scaling (NMDS), distance-based Redundancy Analysis (dbRDA), and Permutational Multivariate Analysis of Variance (PERMANOVA)] revealed clear community structuring primarily driven by salinity, temperature, vegetation type, sampling year, and spatial distance (all *p* < 0.05). eDNA detected 16 conservation-priority taxa listed on the Japan Red List [Critically Endangered (CR): 4, Endangered (EN): 4, Vulnerable (VU): 6, and Near Threatened (NT): 2] and six International Union for Conservation of Nature (IUCN) Red List species (EN:2, VU:4), including *Anguilla japonica* and *Cheilinus undulatus*. Unexpected detections of deep-sea taxa likely reflect tidal transport or diel migrations. Compared with previous checklists by Suzuki & Seno (2005), this study detected additional taxa but not all recorded species, indicating that fish biodiversity in the Urauchi River system remains incompletely characterized and highlighting the importance of integrating eDNA with conventional surveys for a comprehensive assessment. Nevertheless, our study provides the first molecular baseline of fish diversity in the Urauchi River, demonstrating a complementary, non-invasive biodiversity assessment in dynamic mangrove estuaries. Routine eDNA monitoring, combined with multi-seasonal and depth-stratified sampling, provides a practical early warning system to detect ecological shifts and inform targeted conservation and management actions in this globally significant watershed.

## Introduction

The UNESCO designation of World Natural Heritage Sites reflects a global initiative to safeguard areas of outstanding universal value, including regions with vital habitats that support remarkable species richness and threatened taxa (UNESCO, 2024). However, several sites face ongoing conservation challenges, such as habitat degradation, invasive species, anthropogenic pressures from tourism and coastal development, and the lack of sustained and systematic biodiversity assessments (Eken et al., 2004; Jones et al., 2018). These challenges underscore the need for robust, long-term biodiversity monitoring to inform evidence-based management and policy decisions, which allow early detection of ecological changes and objective evaluation of management strategies (Eken et al., 2004; UNESCO, 2024; Jones et al., 2018). Hence, implementing sustainable monitoring frameworks is not only essential for safeguarding the ecological integrity of these sites but also critical for meeting international conservation obligations and enabling effective ecosystem management in the face of environmental change (Thorsell & Hogan, 2014; Pereira et al., 2013; UNESCO, 2024).

The Urauchi River, situated within the World Natural Heritage site of Iriomote Island in Okinawa Prefecture, is widely recognized as one of Japan’s most fish-species-rich rivers (Suzuki & Seno, 2005). The river supports over 400 fish species, including several endangered and rare taxa (Ministry of the Environment, 2020; Suzuki & Seno, 2005; Suzuki & Mori, 2016a), which suggests that the river is the most iconic biodiversity hotspot for ichthyofauna on Iriomote Island (Japan Tourism Agency, 2022). Stretching 18.8 km, it is the longest river in the Ryukyu Archipelago, which was inscribed in 2021 under Criterion (x) for its exceptional biodiversity. Subtropical natural forests surround the river’s upstream segments, while its mid- to downstream reaches form a well-developed mangrove estuarine zone. The basin remains uninhabited, with abundant flow and deep water reaching 15 m in places. Although the river is protected under national and regional conservation frameworks, long-term and sustainable monitoring of fish communities is urgently needed, under the increasing impacts of climate change and anthropogenic activities like coastal development and infrastructure expansion (Japan Tiger and Elephant Fund & Yamaneko Patrol, 2019; Okuda, 2005; Suzuki & Mori, 2016b). However, baseline knowledge of species distribution patterns and the ecological processes shaping this remarkable fish diversity remains limited. Assessing biodiversity in such a dynamic environment with extensive mangrove habitats presents significant challenges due to data gaps, unstandardized methods, and spatial heterogeneity (Chiarucci, Bacaro & Scheiner, 2011; Feller et al., 2010; Lee et al., 2014).

Environmental DNA (eDNA) is increasingly used for aquatic biodiversity, providing a high-resolution approach for characterizing fish communities across complex river–estuary systems (Thomsen & Willerslev, 2015; Deiner et al., 2017). Studies across diverse geographic regions demonstrated its strong performance in detecting riverine biodiversity, often outperforming or complementing traditional methods in taxonomic coverage and sensitivity (Hallam et al., 2021; Poyntz-Wright et al., 2024; Zainal Abidin et al., 2022). Since the development of MiFish primers (Miya et al., 2015), fish eDNA metabarcoding has been widely adopted in freshwater, estuarine, and marine ecosystems (Ahn et al., 2020; Blackman et al., 2021; Naputo et al., 2024; Zainal Abidin et al., 2022; Zou et al., 2020) due to its non-invasive sampling, ease of field implementation, and ability to generate comprehensive, objective species-level data (Barnes & Turner, 2016; Deiner et al., 2017; Huang et al., 2022; Ushio et al., 2018). For fish biodiversity observation and monitoring in the Urauchi River, environmental DNA (eDNA) metabarcoding offers a feasible and scalable tool. In the recent UNESCO-led eDNA expeditions, the deployment of a standardized eDNA-based approach across diverse marine World Heritage Sites demonstrated efficient biodiversity assessment and produced valuable datasets imperative for conservation planning and monitoring (Suominen et al., 2024).

In this study, we apply fish eDNA metabarcoding using surface water samples collected in two separate years in the Urauchi River. Our objectives are: (1) to characterize fish species diversity across the river, from freshwater to estuarine zones; (2) to detect endangered and rare species; (3) to examine fish community composition alongside with environmental variables; and (4) to provide eDNA-based biodiversity data for long-term biodiversity monitoring, conservation planning, and management in this ecologically significant and globally recognized Natural Heritage Site.

## Materials & Methods

### Study area and Sample collection

**Figure 1:**
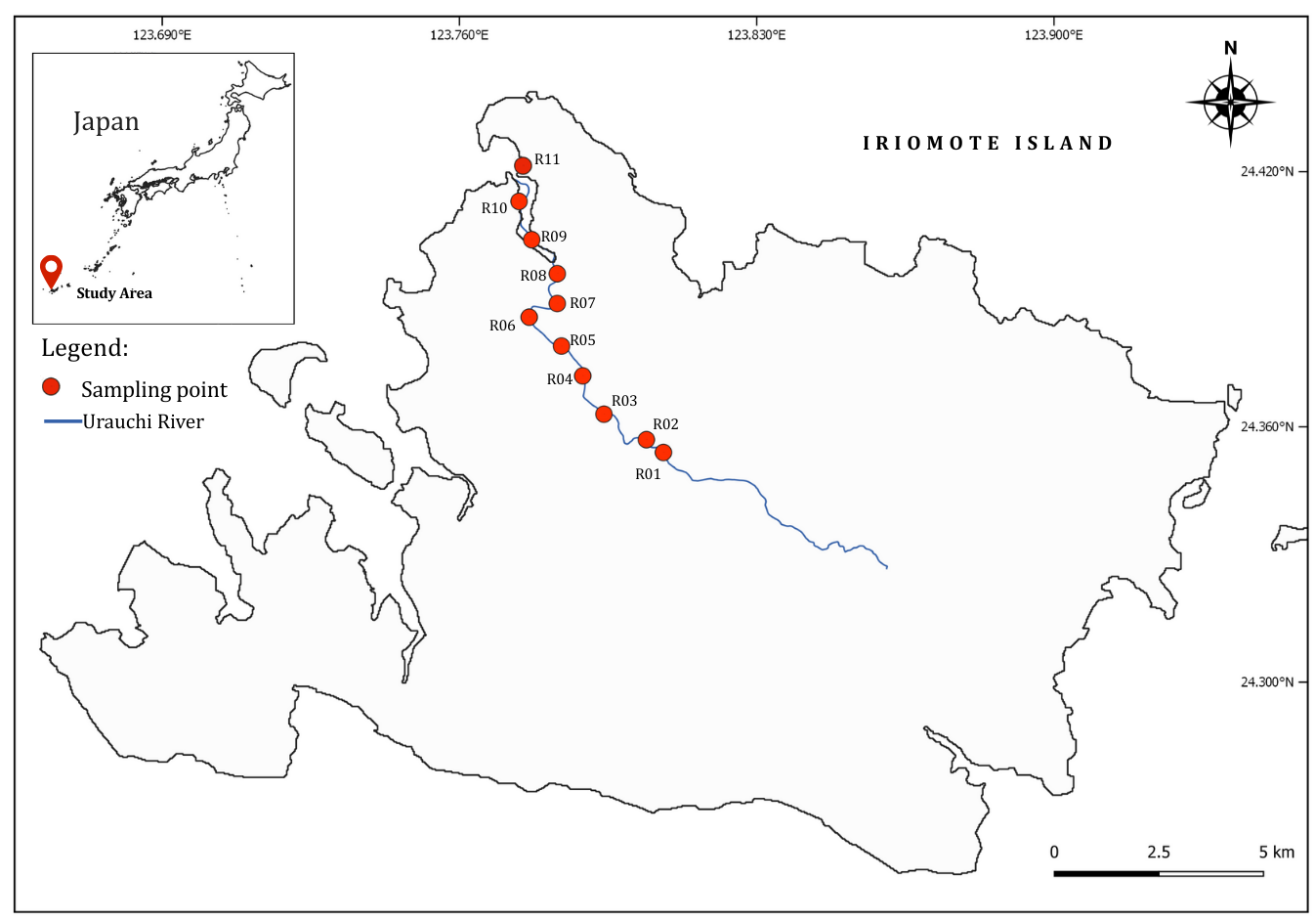
Map of the Urauchi River on Iriomote Island, Okinawa, Japan, showing the 11 sampling sites (R01–R11) surveyed in this study. Red circles indicate sampling locations along the river. The blue line represents the main stream of the Urauchi River. The inset shows the location of Iriomote Island within Japan. The map was created using QGIS version 3.14.

The study site is the Urauchi River located at the Northern end of Iriomote Island (Fig. 1). Located in a subtropical climate zone, the island experiences an annual mean temperature ranging from 17.4–29.2 °C in 2021 and 18.7–30 °C in 2024 (Japan Meteorological Agency, 2025). This river is distinguished by a transition from freshwater to a brackish environment with dense mangrove forest at estuarine sites. Dominant mangrove species included *Bruguiera gymnorrhiza* (prevalent in midstream sites R04–R09), *Rhizophora stylosa* (lower reaches of the river), and additional taxa such as *Kandelia obovata, Avicennia marina*. We conducted water sampling along a 10–11 km stretch of the river, from the mouth to the upstream waterfall (Fig. 1), following the protocol outlined in Environmental DNA Sampling and Experiment Manual by Minamoto et al. (2021). Sampling was carried out at 10 sites in May 2021 and 11 sites in November 2024, with roughly 1 km intervals between sites (Table 1). The additional site (R11) was included in the 2024 survey at the river mouth to capture comprehensive fish community composition along the freshwater–marine interface. Sampling sites, including upstream stations, were located away from residential buildings, hotels, and drainage outlets. Field permits were obtained from the Okinawa District Forest Office, Forestry Agency, Japan with No. 2026 and No. 2085 (for 2021) and No. 6-1-24 (for 2024). Before sampling, the collection buckets and adjustable reach pole water sampler were washed and decontaminated using bleach, followed by thorough rinsing with running freshwater. Sterile gloves were worn and changed at every sampling location to prevent cross-contamination. Approximately 400 mL of surface water was collected ten times from random locations within a 5–10 m radius at each site and pooled in a 5-liter bucket. Subsequently, 900–1,000 mL of water volume was drawn with a sterile 50 mL syringe and filtered through a 0.45 µm Sterivex cartridge (EMD Millipore Corp., USA); two replicates per site were preserved in two mL of DNAiso reagent (TaKaRa Bio, Tokyo, Japan). A field blank was collected on each sampling day and prepared using purified water, following the protocol of Minamoto et al. (2021). It was processed using the same procedures and handled alongside the eDNA water samples. All samples were stored at −20 °C until processing at the Tropical Biosphere Research Center (TBRC). Environmental variables were selected based on their known roles as key abiotic and spatial drivers of estuarine fish communities, with temperature and salinity acting as primary physiological filters and vegetation, elevation, and distance from the sea representing habitat complexity and longitudinal connectivity gradients (Barnes & Turner, 2016; Sildever et al., 2023). At each site, salinity and water temperature were measured using a conductivity meter (Mother Tool Co., Ltd., Taichung City, Taiwan) and a digital thermometer (Tanita Co., Ltd., Tokyo, Japan). Vegetation type was recorded categorically, while elevation and distance to the sea were calculated from GPS coordinates. Photographic documentation was also conducted to support habitat assessment.

**Table 1:** Summary of sampling sites and associated environmental variables, including geographical coordinates (longitude and latitude), location, dominant vegetation, elevation, distance from the sea, water temperature, salinity, and water volume filtered.

Site	**Longitude**	**Latitude**	**Location**	**Vegetation**	**Elevation (m)**	**Dist. from sea (m)**	**Water Temp (°C)**	**Salinity (%)**	**Vol. (ml)**
							2021:2024		
R01	123.80773	24.35419	River	SF	91.8	10.285	23.6:23.3	0:0	1,000:1,000
R02	123.80369	24.35703	River	SF	64.1	9.706	24.1:23.2	0: 0	1,000:1,000
R03	123.79371	24.36346	Upper est.	SF	28.8	8.061	24.4:23.2	0.17:0	1,000:1,000
R04	123.78933	24.37184	Upper est.	SFpm	10	6925	25.1:23.8	0.58:0.01	900:1,000
R05	123.7842	24.37883	Upper est.	SFpm	13.1	5936	24.8:24.1	0.71:0.01	900:1,000
R06	123.7764	24.38588	Middle est.	MFsf	14.5	4.792	25.6:23.9	1.46:0.02	1,000:1,000
R07	123.78317	24.38865	Middle est.	MFsf	9	3,868	24.9:24.7	1.25:0.25	900:1,000
R08	123.78329	24.39584	Middle est.	MFsf	12	2,854	25.3:25.2	2.56:0.67	1,000:1,000
R09	123.77394	24.41257	Lower est.	MF	2.2	1,746	25.7:27.0	3.28:2.43	1,000:1,000
R10	123.7774	24.40407	Lower est.	MFbf	1.1	703	24.1:27.9	3.01:3.29	1,000:1,000
R11	123.7732	24.4186	Lower est.	MFbf	0	0	–:28.3	–:3.32	–:1,000

**Notes.**

est., estuary; Dist., distance; Vol., volume; SF, Subtropical Forest; SFpm, Subtropical Forest with patchy mangroves; MFsf, Mangrove Forest with subtropical forest; MF, Mangrove Forest; MFbf, Mangrove Forest with beach forest.

### eDNA extraction, amplification, and library preparation

Before proceeding from DNA extraction to library preparation, equipment and materials were decontaminated using a sodium hypochlorite solution and exposed to UV light for 20 min before each experimental procedure. To prevent cross-contamination, pre-polymerase chain reaction (PCR) and post-PCR processes were conducted in separate designated areas, each equipped with dedicated tools and materials. The laboratory floor and bench tops were decontaminated routinely with bleach before starting experiments. Access to the clean room was limited to trained personnel, who wore dedicated lab coats, gloves, hair nets, and face masks. Additionally, DNA AWAY Surface Decontaminant (Thermo Scientific) was used to thoroughly clean work surfaces before use.

Environmental DNA (eDNA) samples and field blanks were extracted from Sterivex filters using the DNeasy PowerWater Sterivex Kit (QIAGEN, Hilden, Germany), following the manufacturer’s protocol with minor modifications, and processed with a QIAvac 24 Plus vacuum manifold. DNA was eluted in 200 µL of Solution EB and stored at −20 ^∘^C. We used QIAvac 24 Plus vacuum manifold. To assess contamination risk, a negative extraction control was included. DNA concentrations were quantified using the QuantiFluor ONE dsDNA System High Sensitivity (HS) Assay Kit with a Quantus Fluorometer (Promega). Library preparation was performed using a two-step PCR targeting the mitochondrial 12S rRNA gene’s mid-region (Minamoto et al., 2021; Miya et al., 2015), using MiFish universal primer sets (MiFish-U, MiFish-U2, and MiFish-E-v2 in a 2:1:1 ratio, five pmol/µL). First-round PCR reactions (12 µL total volume) contained six µL of KAPA HiFi HotStart ReadyMix (Kapa Biosystems, Wilmington, MA, USA) or PrimeSTAR Max DNA Polymerase (Takara Bio Inc., Ohtsu, Shiga, Japan), 2.8 µL of primer mix, 1.2 µL of UltraPure distilled water (Invitrogen, Waltham, MA, USA), and two µL of template DNA. Reactions were prepared in quadruplicate for each enzyme using an epMotion 5070 automated pipetting system (Eppendorf, Hamburg, Germany). A PCR-negative control using UltraPure distilled water was included. The first-round PCR thermal cycling conditions were as follows: initial denaturation at 95 ^∘^C for 3 min; 35 cycles of denaturation at 98 ^∘^C for 20 s (KAPA) or 10 s (PrimeSTAR), annealing at 65 ^∘^C (KAPA) or 63 ^∘^C (PrimeSTAR) for 15 s, and extension at 72 ^∘^C for 15 s; followed by a final extension at 72 ^∘^C for 5 min.

PCR products were then visualized on a 2% agarose gel prepared with 0.5× TAE buffer, stained with Midori Green (Nippon Genetics, Tokyo, Japan), and run at 135 V for 30 min using a gel electrophoresis chamber. PCR amplicons were combined and purified using the NucleoMag NGS Clean-up and Size Select Kit (MACHEREY-NAGEL GmbH and Co. KG, Duren, Germany). DNA concentrations were normalized by measuring band intensities on agarose gels using ImageJ software, following a densitometric method (Ohgane & Yoshioka, 2019). Second-round PCR (15 µL total volume) included 1.76 µL of dual-index Nextera adapters (Macrogen), 3.88 µL of UltraPure water, and 1.86 µL of normalized first-round PCR product. The PCR profile was: initial denaturation at 95 ^∘^C for 3 min; 10 cycles of 98 ^∘^C for 20 s and 72 ^∘^C for 15 s; and a final extension at 72 ^∘^C for 5 min. Amplicon size (∼370 bp) was verified with the Qsep1-Lite Bio-Fragment Analyzer (BiOptic, Taipei, Taiwan). Final libraries were combined, size-selected using 2% E-Gel SizeSelect II Agarose Gels (Invitrogen, Thermo Fisher Scientific), purified, and quality-checked with the Qsep1-Lite system. Sequencing was performed on an Illumina NovaSeq 6000 platform (PE150) by Novogene, coordinated through the Gigayomi sequencing service (Nippon Genetics Co., Ltd., Tokyo, Japan).

### Bioinformatics

Raw paired-end eDNA sequences were processed with *fastp* (Chen et al., 2018) to eliminate low-quality reads. Qualified reads were imported into QIIME 2 v2024.2 (Bolyen et al., 2019) for downstream processing. Primer sequences were trimmed using Cutadapt (Martin, 2011) within the *q2-cutadapt* plugin. Sequence quality was assessed with the *q2-demux* summarize plugin. Based on quality profiles, denoising, chimera removal, and amplicon sequence variant inference were performed using DADA2 (Callahan et al., 2016) implemented in the *q2-dada2* plugin. Amplicon sequence variants were clustered into operational taxonomic units (OTUs) at 99% sequence identity using VSEARCH (Rognes et al., 2016) in *de novo* mode through the *q2-vsearch* plugin. Feature and sequence metadata were visualized using the *q2-metadata* plugin. All analyses were performed using QIIME 2 within a conda environment. OTUs were searched against the NCBI Nucleotide BLAST (blastn) database (Zhang et al., 2004), and phylogenetic trees for each sequence were generated using FastTree (Price, Dehal & Arkin, 2010). All QIIME2 commands and phylogenetic tree parameters (Supplementary Materials S1 and S2) used in this study are deposited in an external repository on the Open Science Framework (OSF; https://osf.io/p3wx7/).

### Taxonomic assignment of OTUs

Given the lack of a globally curated fish eDNA reference database, each sequence was assessed individually on a case-by-case basis (Table S1). Sequences with low read counts (≤10) that occurred only once were automatically discarded. A sequence identity threshold of ≥98.5% was initially considered for species-level assignment (Naputo et al., 2024). However, taxonomic assignment was accepted only for sequences clustering within a single-species clade, after thorough assessment using criteria such as phylogenetic tree placement, expert taxonomic input, and comparison with a reference species list compiled from local faunal surveys and the national checklist of Japanese fishes. When a sequence matched with multiple species, it was conservatively annotated at the genus level and labeled as “sp”. until further resolution. Sequences falling within clades that included multiple genera of the same family were assigned at the family level. If sequences could not be annotated even at the family level, we categorized them as “unannotated OTU”. Sequences showing only minor base pair differences (1–2 bpd) but matching the same reference species in NCBI, and appearing in a unique sample or at least two samples supported by read-abundance patterns, were classified as putative haplotypes (*e.g.*, species1, species2), while closely related lower-read sequence variants within the same sample were treated as likely sequencing errors and excluded.

OTUs representing non-endemic species and unlikely to occur naturally in the Ryukyu Islands were excluded unless the corresponding genus is known to occur in Japan. However, deep-water species with relevant occurrence records and frequent detection with high read abundance were retained, due to the potential influence of dynamic ocean currents. Taxonomic names were based primarily on the Catalog of All Japanese Fish Species Online ver. 38. (Motomura, 2026), which was used to validate annotated OTUs. Habitat classification of annotated OTUs was performed using the rfishbase R package (Boettiger, Lang & Wainwright, 2012), which accesses FishBase (Froese & Pauly, 2024) for ecological and habitat information, with additional reference to Fishes of the World (Nelson, 2006).

### Data handling and analysis

As part of the quality control process, six negative controls (field blank, extraction blank, and PCR negative controls) were included during library preparation. Of these, only one, associated with a single enzyme, yielded low-level reads, representing approximately 0.35% of the total reads in the corresponding sample. This was considered a potential source of contamination, as it was the only case in which a negative control and its associated sample shared a similar pattern of fish occurrence. These reads (ranging from 9 to 356) were interpreted as potential contamination (*e.g.*, due to proximity during handling) and were conservatively excluded from downstream analyses by subtracting them from all possible affected samples.

To evaluate species richness and community composition across sites, alpha and beta diversity metrics were computed. eDNA read counts were Hellinger transformed (*i.e.,* relative abundance followed by square root transformation) to normalize for sequencing depth reads (Legendre & Gallagher, 2001). Alpha diversity indices (Shannon and Simpson) and rarefaction curves illustrating OTU richness across samples with increasing sequencing depth were generated using the phyloseq R package (McMurdie & Holmes, 2013). OTU abundance patterns and site similarity were visualized using bar plots, heatmaps, and dendrograms. Community composition analyses focused on OTUs present in ≥30% of total samples with high read abundances, using this threshold as a conservative filter to retain spatially consistent taxa and reduce noise, sequencing artifacts, and singletons. Beta diversity was assessed using Bray–Curtis dissimilarity and visualized *via* non-metric multidimensional scaling (NMDS) to check variation in fish community composition among sites, as this ordination method is well-suited for non-linear ecological data (Oksanen et al., 2025). Relationships between fish community structure and measured environmental variables were investigated through distance-based redundancy analysis (db-RDA) to identify environmental drivers of the observed compositional patterns (Legendre & Anderson, 1999). The significance of the overall model, individual axes, and marginal effects of predictors was evaluated using permutation tests (999 iterations). Additionally, a PERMANOVA (Anderson, 2017) with Bray–Curtis dissimilarities was performed to test for differences in community composition across categorical groups and environmental variables. All visualizations and statistical analyses were conducted in R version 4.2.3 (R Core Team, 2023), primarily using the vegan package (Oksanen et al., 2025) for multivariate analyses and ggplot2 (Wickham, 2016) for data visualization. Figures were modified for presentation clarity using the Canva online design platform (https://www.canva.com).

## Results

### eDNA fish data overview

**Figure 2:**
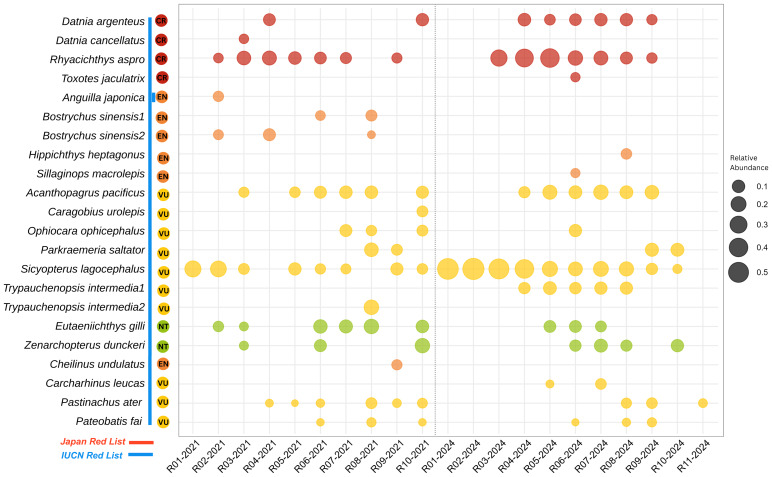
Detection of conservation-priority fish species in the Urauchi River based on environmental DNA metabarcoding. Species are categorized according to their conservation status on the Japan Red List (Ministry of the Environment, 2020; orange lines) and the IUCN Red List (International Union for Conservation of Nature (IUCN), 2025; blue lines), with status indicated as CR (Critically Endangered), EN (Endangered), VU (Vulnerable), NT (Near Threatened), or LC (Least Concern). Relative abundance is represented by the size of each circle, calculated from Hellinger-transformed OTU read counts averaged across enzymes, samples, and years for each site.

A total of 42 water samples were obtained and sequenced from 11 sites along the Urauchi River. After filtering, denoising, and merging paired-end reads, 35.7 million reads were obtained from target taxa (Table S1). OTU richness across samples with increasing sequencing depth is shown in Fig. S1. Using the NCBI reference database and other relevant sources, and following thorough evaluation and verification, we identified 332 Operational Taxonomic Units (OTUs) distributed across the sampling sites (Table 2). Our results detected the presence of two classes (Actinopterygii and Chondrichthyes), comprising 30 orders (dominated by Gobiiformes), 73 families, and 177 genera. Of the total OTUs detected, 254 (76%) were assigned to the species level, among the remaining OTUs, 64 (19%) to the genus level, 11 (3%) to the family level, and three (1%) were not assigned to any of them and treated as unannotated OTU (Table 2). Of the OTUs assigned to species, four were classified as CR, four as EN, six as VU, and two as NT according to the Japan Red List (Ministry of the Environment, 2020), while the International Union for Conservation of Nature (2025) categorized two species as EN and four as VU (Fig. 2). Comparison with Suzuki & Seno’s (2005) checklist showed that eDNA detected both overlapping and distinct taxa. At the species level, while approximately 146 species from the previous checklist were detected by the present eDNA study, 87 species were not in the prior list (Table 3: Table S2). Similar trends were observed at the genus and family levels.

**Table 2:** Summary of OTU annotation by taxonomic rank. A total of 332 OTUs detected by eDNA metabarcoding were annotated to the family, genus, or species levels. OTUs assigned at the family and genus levels could not be resolved to lower taxonomic ranks. Species-level assignments included 23 OTUs representing 11 species with identified haplotypes. Three OTUs remained unannotated due to insufficient taxonomic resolution

**Annotation**	**OTUs annotated**
Family	11
Genus	64
Species	254
Unannotated	3

**Table 3:** Comparison of taxonomic groups detected by eDNA metabarcoding (excluding deepwater species and unannotated OTUs) and those recorded in the checklist published by Suzuki & Seno (2005). Taxonomic names were standardized using All Japanese Fish Species Online, ver. 38 (Motomura, 2026). Species-level identifications from the checklist are detailed in Table S2.“eDNA” denotes the total number of taxa detected using the eDNA method, with haplotypes of the same species counted as a single taxon; “eDNA only” refers to taxa detected exclusively by eDNA; “Checklist” denotes the total number of taxa reported in the previous study; “Checklist only” refers to taxa recorded only in the previous study; and “Both” denotes taxa detected by both methods.

	**eDNA**	**eDNA only**	**Checklist**	**Checklist only**	**Both**
Family	70	11	84	28	59
Genus	170	40	220	90	130
Species	233	87	371	225	146

### Detection of species with higher conservation priority

Our eDNA study detected the presence of several species with higher conservation priority. Sixteen species of conservation categories were listed in the Japan Red List (Ministry of the Environment, 2020) (Fig. 2). Twelve species were detected in both sampling years, with some species uniquely detected each year. In comparison to the IUCN Red List 2025 of Threatened Species, two species were listed as EN, four as VU, with two species only detected in 2021, while vulnerable stingray species (*P. ater*, *P. fai*) were detected in both years. The bull shark (*C. leucas*), known for its ability to traverse marine and freshwater systems, was detected only in 2024 at the intermediate sites. Aside from the detected threatened fish species, deep water (3%), and unannotated fish OTUs (1%) were recorded.

### Fish diversity and assemblage along the Urauchi River

**Figure 3:**
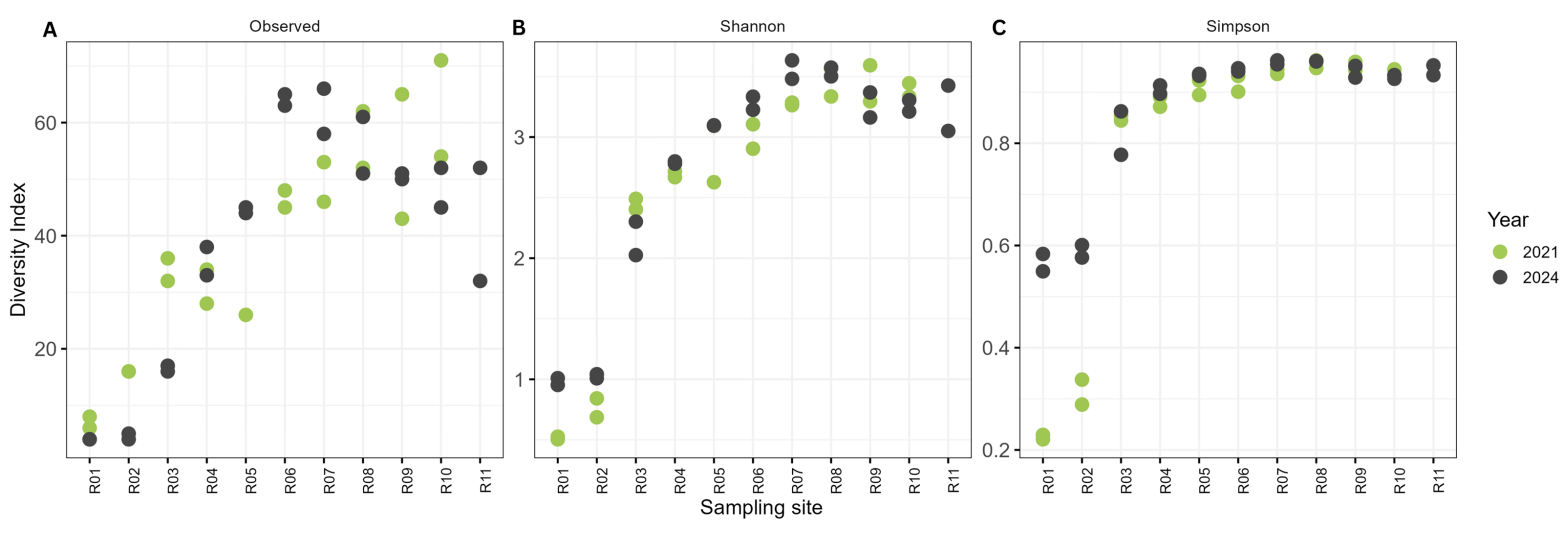
Fish diversity at sampling sites along the Urauchi River, Iriomote Island. (A) Observed species richness, (B) Shannon diversity index, and (C) Simpson diversity index were calculated based on two replicate samples per site, each processed with two restriction enzymes, from surveys conducted in 2021 and 2024. Green and black dots indicate values from 2021 and 2024, respectively.

**Figure 4:**
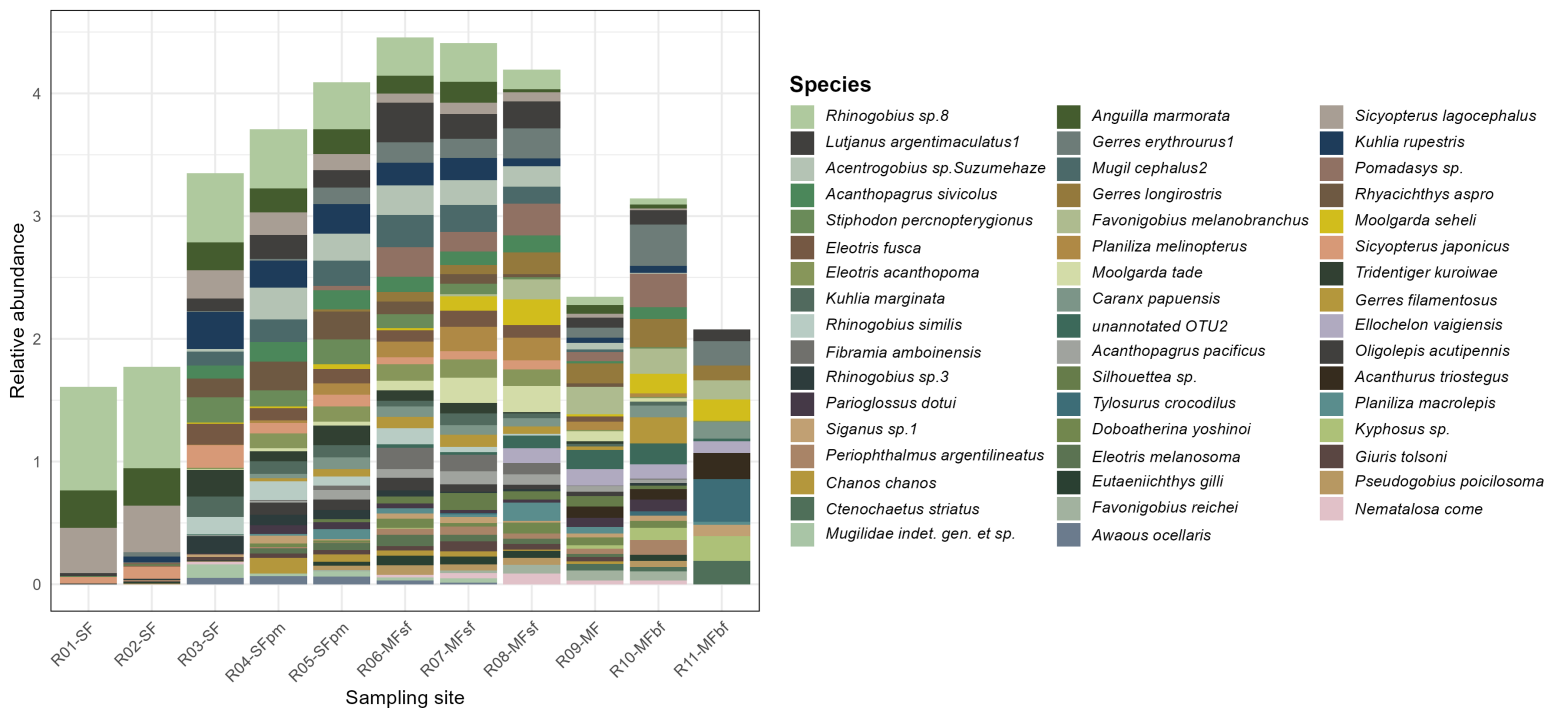
Fish community composition based on major taxa at sampling sites along the Urauchi River, Iriomote Island. Relative abundances were calculated from Hellinger-transformed read counts averaged across enzymes, replicates, and years. Site vegetation types are classified as follows: SF –Subtropical Forest; SF-pm, Subtropical Forest with patchy mangroves; MFsf, Mangrove Forest with adjacent subtropical forest; MF, Mangrove Forest; MFbf, Mangrove Forest with beach forest.

Fish diversity and assemblage across the Urauchi River were shown in the 2021 and 2024 surveys, which suggests distinct assemblages from upstream sites (R1–R3) to estuarine sites (R4–R11). The upstream site R1 consistently demonstrated the lowest OTU richness in both years. In contrast, the highest number of OTUs was recorded at the lower estuary R9–R10 in 2021, while the 2024 survey identified a peak at the mid-estuary site R6–R7 (Fig. 3A). Alpha diversity indices (Shannon and Simpson) followed similar patterns, gradually increasing from upstream freshwater habitats toward estuarine and coastal zones (Figs. 3B, 3C). Across samples collected in different years, major fish species were identified based on their prevalent occurrence and high read abundance (Fig. 4; Fig. S2).

### Environmental influences on fish assemblages

**Figure 5:**
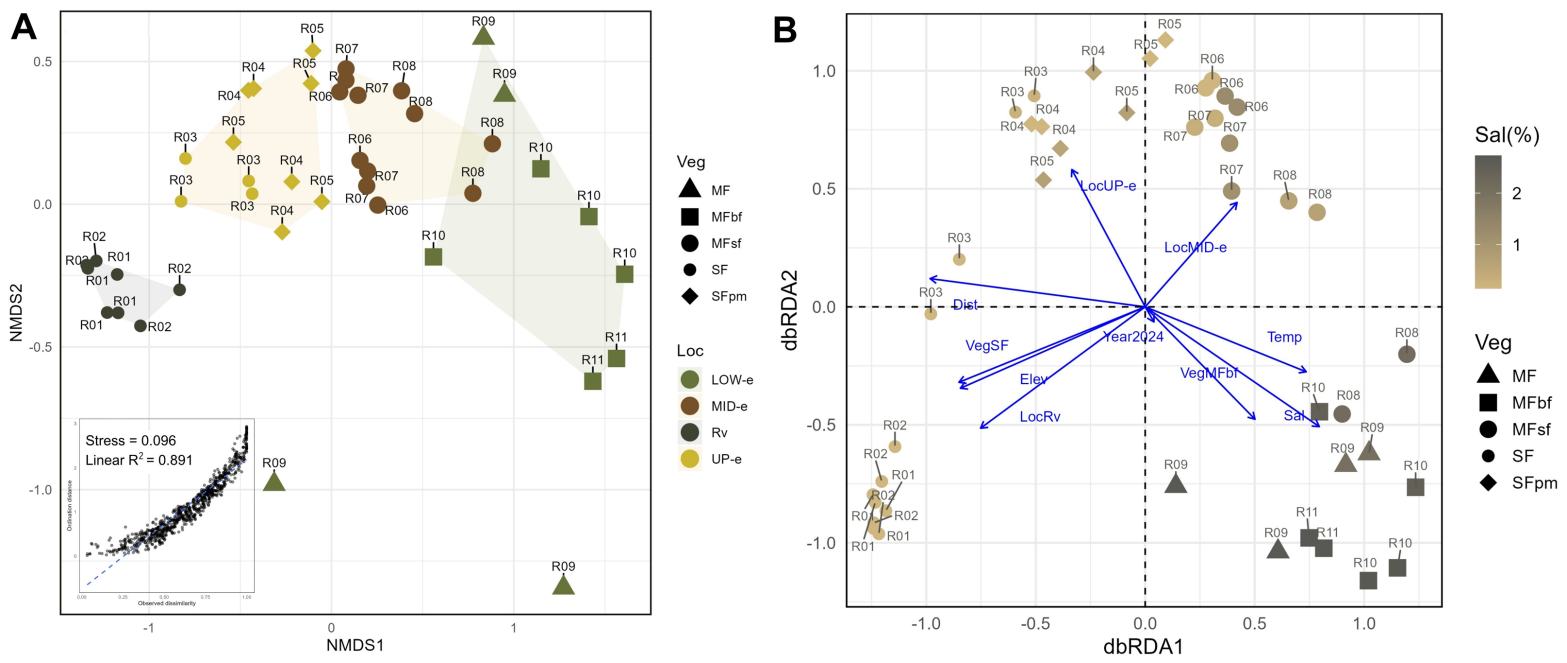
Multivariate ordination of fish community structure based on eDNA data from the Urauchi River. Each point represents one replicate sample processed with one of two enzymes, across surveys from 2021 and 2024. (A) Non-metric multidimensional scaling (NMDS) plot illustrating fish community composition across sampling sites. Shapes represented by vegetation type - Subtropical Forest (SF), Subtropical Forest with patchy mangroves (SF-pm), Mangrove Forest with subtropical forest (MFsf), Mangrove Forest (MF), and Mangrove Forest with beach forest (MFbf). Points are colored by sampling location: river (RV), upper estuary (UP-e), middle estuary (Mid-e), and lower estuary (Low-e). (B) Distance-based redundancy analysis (db-RDA) biplot showing relationships between fish communities and environmental variables. Blue arrows indicate significant predictors: elevation (Elev), distance from the sea (Dist), vegetation type (Veg), water temperature (Temp), and salinity (Sal). Sampling sites (R01–R11) are colored by salinity and shaped by vegetation as in (A).

Multivariate analyses of eDNA-derived fish diversity data showed distinct fish assemblages across both sampling sites and years. NMDS ordination with a stress value of 0.096 confirmed a reliable two-dimensional representation (Fig. 5A). Upstream river sites (R01–R02) clustered tightly, while estuarine sites (R05–R11) showed increasing differentiation in communities, with R09 (2021) more dispersed relative to other sites (Fig. 5A). Cluster dendrogram based on Bray–Curtis dissimilarity analyses supported this clustering of fish communities, particularly in same site locations (river, upper-middle-lower estuarine sites) (Fig. 5A; Fig. S3). Both distance-based redundancy analysis (dbRDA) and PERMANOVA tests on the top-occurring species with the highest eDNA proportional reads captured key variation in species patterns along environmental gradients. The distance-based redundancy analysis (dbRDA) model was highly significant overall, explaining 77.62% of the variation in species composition (*F* = 10.72, *p* = 0.001), with dbRDA1 and dbRDA2 accounting for 47.4% and 23.1% of constrained variation , as illustrated in the db-RDA ordination plot (Fig. 5B). Sites R09–R11, located in the lower estuary, were associated with higher salinity, elevated temperature, and dense mangrove vegetation. Sites R06–R08, influenced by their middle estuary location, and sites R03–R05, shaped by upper estuary conditions, exhibited intermediate community composition, reflecting a transitional zone along the estuarine gradient. Upstream riverine sites (R01–R02), characterized by increasing elevation and distance from the sea, correspond to freshwater conditions and subtropical forest vegetation. The first five dbRDA axes were significant (*p* < 0.01), capturing major species variation along environmental gradients. Furthermore, marginal tests of individual environmental variables within the dbRDA showed significant effects of Salinity (*F* = 3.52, *p* = 0.003), Vegetation (*F* = 2.75, *p* = 0.006), Year (*F* = 6.41, *p* = 0.001), Distance (*F* = 2.52, *p* = 0.030), and Temperature (*F* = 4.41, *p* = 0.001). Location showed a marginal effect (*F* = 2.07, *p* = 0.062), and Elevation was non-significant (*F* = 0.33, *p* = 0.973). Complementary PERMANOVA results also identified Salinity (*F* = 3.61, *p* = 0.004), Vegetation (*F* = 2.98, *p* = 0.008), Year (*F* = 4.65, *p* = 0.002), Distance (*F* = 2.55, *p* = 0.036), and Temperature (*F* = 4.80, *p* = 0.001). Overall, both spatial and environmental variables play important roles in shaping species distributions and community structure.

## Discussion

### Remarkable fish diversity of the Urauchi River detected by eDNA metabarcoding

MiFish eDNA metabarcoding analysis revealed that the Urauchi River, located within a UNESCO World Natural Heritage Site, harbors exceptionally high fish species diversity. In this study, we detected 332 OTUs from surface water samples. This number is lower than the ∼407 species checklist recorded from 30 years (1974–2005) conventional taxonomic surveys (Suzuki & Seno, 2005). The discrepancy may be attributed to methodological differences and the inability to resolve taxa to the species level, together with inherent limitations of eDNA approaches, including variable DNA shedding rates and transport, PCR primer selectivity, and incomplete reference databases (Zainal Abidin et al., 2022; Kelly, Shelton & Gallego, 2019; Miya, 2022; Harrison, Sunday & Rogers, 2019). Specifically, our eDNA survey was limited to surface water across two sampling events only and thus likely underrepresented benthic, substrate-associated, deep-dwelling, seasonally active, and nocturnal species included in the checklists. Nevertheless, about 146 species from the previous checklist were detected in this eDNA study, while the 87 OTUs annotated at the species level, excluding deep-sea taxa detected, were not recorded in the previous checklist (Table 3: Table S2). The number of newly detected species, when combined with the 371 classified at the species level from the previous checklist, accounts for an estimated 458 total species. The number of species used for this estimate is only 254 among the total 332 detected OTUs. Approximately 34 fish species in the previous checklist were not identified to the species level (Table S2). Although 75 OTUs overlapped at the genus level with these records, the lack of species-level resolution prevents direct comparison. Consequently, estimates of total diversity depend on how unresolved OTUs are treated, particularly given ongoing improvements in reference databases and regional DNA barcode coverage (Deiner et al., 2017; Miya, 2022).

The fact that eDNA data can show such remarkable diversity within just a 10 km stretch of river underscores the ecological uniqueness of the Urauchi River, which surpasses not only other river and coastal systems in Japan (Kume et al., 2021; Yamamoto et al., 2017) but also those in tropical and subtropical regions globally (Zainal Abidin et al., 2022; Blackman et al., 2021; Naputo et al., 2024), as shown by eDNA. This high fish diversity reflects longitudinal connectivity along the freshwater–estuarine–marine continuum (Suzuki & Senou, 2005). Previous long-term surveys documented resident freshwater and estuarine species as well as transient coastal and pelagic taxa, a pattern also detected by eDNA (Table S2). However, tidal transport of marine-derived eDNA is common in estuarine systems and could likely influence the detection of newly recorded putative species (Zainal Abidin et al., 2022; Deiner & Altermatt, 2014; Yamamoto et al., 2017). As eDNA reflects an integrated DNA pool influenced by hydrodynamics and ecological processes, it may not fully resolve resident riverine assemblages. However, combining it with conventional sampling may enable a more comprehensive assessment and refine biodiversity estimates of the incompletely characterized fish diversity of the Urauchi River mangrove estuary.

### eDNA metabarcoding for monitoring fish species with conservation priority

This study demonstrated that eDNA metabarcoding is a useful method for detecting threatened, endemic, and elusive fish species in the Urauchi River, which can provide essential data for ecosystem management and long-term monitoring. From the fish species listed in the 2020 Red List of Japan (Ministry of the Environment, 2020), a total of 16 species were detected, including four Critically Endangered (CR), four Endangered (EN), six Vulnerable (VU), and two Near Threatened (NT) species. Detection patterns were nearly consistent between the two sampling years for the species detected in both years (2021 and 2024) (Fig. 2). Among the species in the previous checklist of the Urauchi River (Suzuki & Mori, 2016a), 43 fish species were listed in the 2020 Red List (CR: 23, EN: 12, VU: 5, NT: 8). Of these, 16 species were detected in this study by eDNA. Several Red List species with low read abundance, such as *Anguilla japonica* (Japanese eel), *Hippichthys heptagonus*, and *Toxotes jaculatrix* (archerfish), were detected, reflecting the sensitivity and effort efficiency of the eDNA method. In addition, six IUCN Red List taxa (EN: 2, VU: 3) were also detected, which include sharks and rays (*C. leucas*, *P. ater*, *P.fai*). The difference in the number of Red List species detected likely reflects several factors, including limited temporal coverage and species-level annotation, exclusive use of surface water samples, and the possibility that rare species may fall below the detection threshold due to low eDNA concentrations (Harrison, Sunday & Rogers, 2019). Although not tested here, previous work suggests that improved sampling strategies (*e.g.*, depth-integrated or benthic water sampling, sediment eDNA, increased sampling frequency, and multiple primers may improve detection of rare or elusive taxa (Deiner et al., 2017; Miya, 2022; Yamamoto et al., 2017).

Unexpectedly, eight OTUs identified as deep-sea taxa (*e.g.*, *Bolinichthys indicus*, *Cyclothone* spp., *Diaphus perspicillatus*, *Lampadena luminosa*) were detected in estuarine samples. These mesopelagic species typically inhabit depths >200 m and are unlikely to occur naturally in riverine environments (Robison, 2004). Their detection likely reflects passive eDNA or organism transport *via* tidal exchange, diel vertical migration through the hydrologically connected mangrove –ocean interface, or secondary ingestion by predators (Catul, Gauns & Karuppasamy, 2011; Harrison, Sunday & Rogers, 2019). Transport of extracellular eDNA or trophic interactions (*e.g.*, prey DNA detected in the feces of migratory predators) may represent plausible mechanisms leading to eDNA detection without the direct presence or active movement of the organism (Bairoliya, Koh Zhi Xiang & Cao, 2022). Similar observations in Okinawan lagoons (Oka et al., 2021) suggest this is not uncommon, though interpretations must be cautious in dynamic systems, particularly in the Kuroshio region. The detection of high-read, unidentified OTUs suggests gaps in reference databases or the existence of undescribed taxa, underscoring the need for further study to fully understand local biodiversity (Miya, 2022; Thomsen & Willerslev, 2015).

### Spatial patterns of fish diversity in the Urauchi River

**Figure 6:**
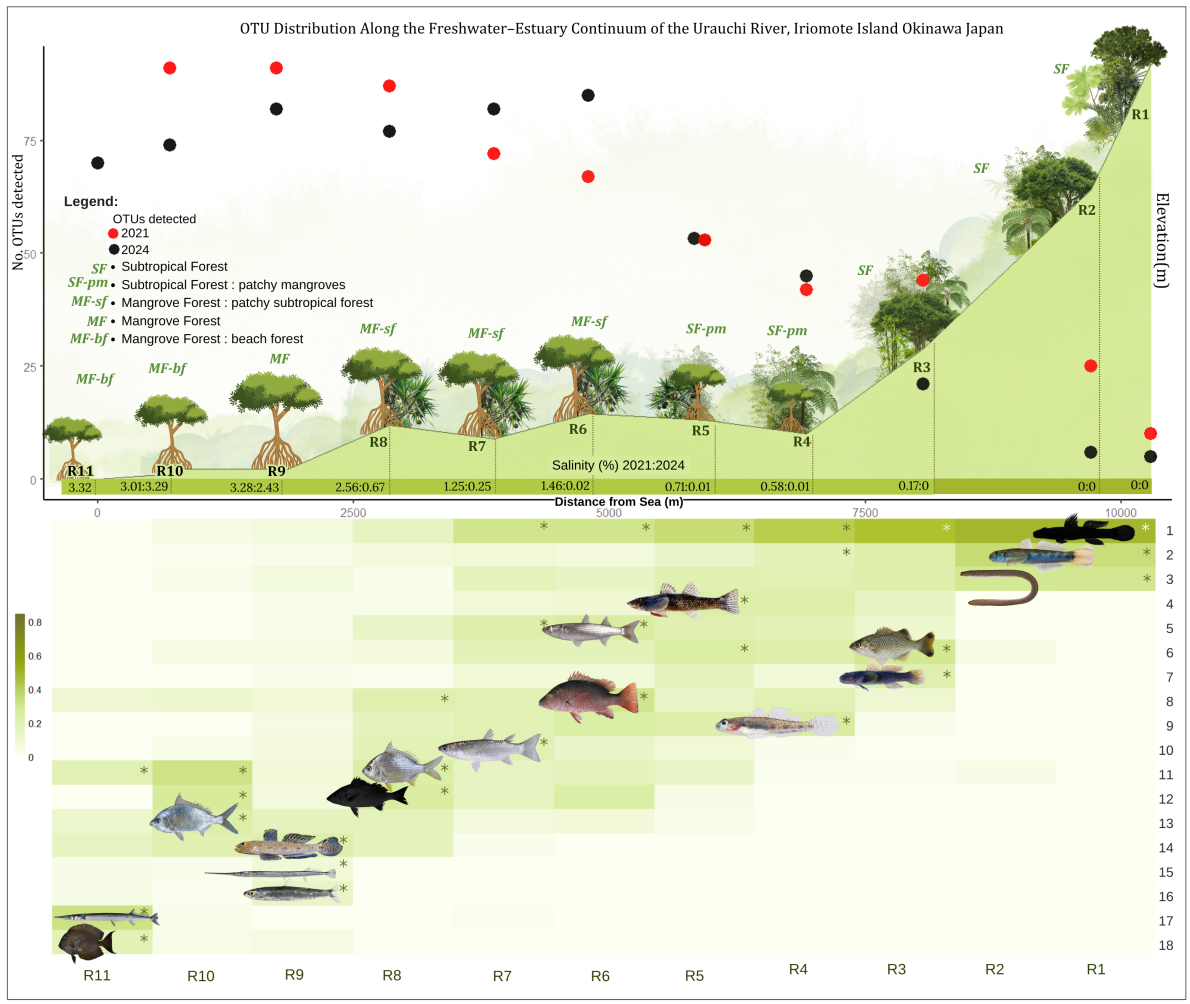
Spatial distribution of fish OTUs along the freshwater-estuarine gradient of the Urauchi River, Iriomote Island, Okinawa, Japan. The upper panel shows the number of OTUs detected at each site in 2021 (red dots) and 2024 (black dots), superimposed on an elevation profile with vegetation types, salinity levels (mean ±SD), and distance from the sea. Vegetation types include: Subtropical Forest (SF), Subtropical Forest with patchy mangroves (SF-pm), Mangrove Forest with subtropical forest (MFsf), Mangrove Forest (MF), and Mangrove Forest with beach forest (MFbf). The lower panel presents a heatmap of the relative eDNA read abundance for 18 dominant fish species detected across sites, based on mean Hellinger-transformed values across samples, enzymes, and years. Asterisks (*) denote species with the highest relative abundance at each site. Species illustrated: (1) *Rhinogobius* sp., (2) *Sicyopterus lagocephalus*, (3) *Anguilla marmorata*, (4) *Rhyacichthys aspro*, (5) *Mugil cephalus*, (6) *Kuhlia rupestris*, (7) *Tridentiger kuroiwae*, (8) *Lutjanus argentimaculatus*, (9) *Acentrogobius* sp. SUZUMEHAZE, (10) *Planiliza melinopterus*, (11) *Gerres erythrourus*, (12) *Pomadasys* sp., (13) *Gerres longirostris*, (14) *Favonigobius melanobranchus*, (15) *Strongylura incisa*, (16) *Spratelloides delicatulus*, (17) *Tylosurus crocodilus*, (18) *Ctenochaetus striatus*. Images reproduced with permission from the Kanagawa Prefectural Museum of Natural History (KPMNH); photographers include T. Suzuki, H. Senou, and A. Murase. Full photo credits are provided in Supplementary Materials S3.

The high fish diversity revealed by our eDNA study in the Urauchi River showed distinct spatial structuring along the river, likely driven by diverse transitional habitats across the freshwater–estuarine gradient. Freshwater species dominated upstream (R1–R2), euryhaline taxa mid-estuary (R3–R9), and marine species near the mouth (R10–R11) (Fig. 6). Gobiidae was the most frequently detected family, with *Rhinogobius* spp. occurring widely, suggesting habitat generalism and adaptability among gobiids (Yamasaki et al., 2015). Freshwater species that were also detected in the downstream reaches are likely due to inflows from upstream transport and numerous tributaries throughout the Urauchi River system (Shogren et al., 2017). Amphidromous species (*e.g.*, *Eleotris fusca*, *Awaous ocellaris*, *Stiphodon lagocephalus*) were primarily detected in up to midstream reaches, reflecting their adaptation to high-flow, oxygen-rich environments (McDowall, 2010). Furthermore, the occurrence of catadromous species (*e.g.*, *Anguilla japonica*, *A. marmorata*) across multiple river sections in our dataset emphasizes the need for unimpeded river–ocean pathways to support long-distance migrations (Able, 2005; Elliott & Whitfield, 2011; Risser, 1995).

Mangrove-associated sites (MF, MFbf, SFpm, MFsf) showed the highest fish diversity, dominated by euryhaline and transitional species (Figs. 4 & 6). These complex habitats function as ecological corridors linking freshwater, estuarine, reef, and pelagic systems (Mumby, 2005; Sheaves, 2005; Sloterdijk et al., 2017). High proportions of eDNA reads assigned to species such as *Lutjanus argentimaculatus*, *Gerres erythorhynchus*, *Kuhlia rupestris*, *Mugil cephalus*, *Acanthopagrus* sp., and *Pomadasys* sp. across multiple samples may indicate that these taxa dominate the local eDNA signal in brackish zones (Figs. 4 & 6). Brackish specialists (*Callogobius tanegasimae*, *Eutaeniichthys gilli*, *Pandaka trimaculata*) and several conservation-priority taxa were also detected. These sites supported herbivores, predators, cryptic, benthic, and juvenile fishes, consistent with previous trophic studies (Nanjo, Kohno & Sano, 2008; Nanjo et al., 2010; Nanjo et al., 2011; Nanjo et al., 2014a; Nanjo et al., 2014b) and reflecting inferred habitat heterogeneity driven by mangrove root complexity, mixed substrates, and salinity gradients that increase niche availability (Lee et al., 2014; Nagelkerken et al., 2008; Pantallano, Bobiles & Nakamura, 2018; Tews et al., 2004). Downstream, marine species such as *Tylosurus crocodilus* and *Ctenochaetus striatus*, as well as reef and pelagic taxa (*Plectropomus leopardus*, *Scomberomorus delicatulus*), further indicate connectivity with adjacent marine systems. These patterns align with the River Continuum Concept (Vannote et al., 1980), supported by empirical studies across fluvial systems (Borges et al., 2020; Brown et al., 2011), where species richness increases downstream due to greater habitat complexity and connectivity (Able, 2005; Sheaves et al., 2015). Diversity indices increased along the freshwater–marine gradient (Figs. 3A–3C), and comparisons between the two sampling years revealed partially overlapping OTU compositions, suggesting some consistency in the taxa detected in the river (Fig. 4; Fig. S2). Collectively, findings highlight mangroves as biodiversity hotspots and key nodes for ecological connectivity, underscoring the conservation importance of the Urauchi River.

### Environmental drivers of fish assemblage structure in the Urauchi River

Multivariate analyses revealed that fish assemblage structure in the Urauchi River is shaped by an interplay of environmental factors. Non-metric multidimensional scaling indicated clear spatial patterns in community composition (Fig. 5A), while distance-based redundancy analysis (dbRDA) and PERMANOVA identified salinity, temperature, vegetation type, sampling year, and spatial distance as significant predictors of assemblage variation (Fig. 5B). These findings align with previous studies, which highlight salinity as a key abiotic filter in estuarine ecosystems, influencing fish distributions through physiological tolerances and habitat suitability (Elliott & Quintino, 2007; Elliott & Whitfield, 2011). The clear zonation of freshwater, brackish, and marine taxa along the river continuum supports the role of abiotic filtering (Borges et al., 2020). Vegetation structure also contributed to community differentiation, following a longitudinal gradient from upstream subtropical forests (R1–R2), through mixed transitional zones (SFpm, MFsf), to downstream mangrove-dominated habitats (MF, MFbf; R9–R11) (Fig. 5A; Fig. S3). An exception was the R09 site in 2021, which may reflect site-specific stochastic variation in eDNA transport and retention within the tidal mangrove zone, rather than local vegetation alone (Harrison, Sunday & Rogers, 2019; Shogren et al., 2017; Deiner et al., 2016). The co-variation of salinity and vegetation suggests that habitat heterogeneity, particularly the presence of complex mangrove architecture, supports a wide diversity of functional groups. Strong correlations among environmental variables may result in confounding effects, where multiple interacting gradients jointly shape fish assemblage patterns (Nagelkerken et al., 2008; Mumby, 2005).

Spatial variables such as river distance and elevation, proxies for hydrological connectivity and fragmentation, also influenced these patterns (Sildever et al., 2023). This conforms with metacommunity theory, which emphasizes the importance of dispersal processes and spatial configuration in riverine biodiversity (Leibold et al., 2004). The community patterns in the Urauchi River likely reflect the interplay between species sorting along environmental gradients and dispersal-driven mass effects (Patrick et al., 2021), supporting the framework that local environmental filters often exert the most significant selective pressure on fish assemblages despite active spatial dispersal processes (Borges et al., 2020). The ecotonal nature of the Urauchi River further promotes species coexistence through edge effects, where transitional habitats support high taxonomic and functional diversity (Risser, 1995). Strong spatial structuring of OTUs in both 2021 and 2024 confirms the utility of eDNA metabarcoding in detecting fine-scale biodiversity patterns. Despite these clear trends, some variation remains unexplained, possibly due to several unaccounted abiotic factors (*e.g.*, turbidity, dissolved oxygen, or nutrients), episodic disturbances, or complex biotic interactions that are likely to affect eDNA distribution and detection (Castellanos-Galindo & Krumme, 2013). Seasonal dynamics likely play a key role as well. Shifts in oceanographic conditions, including declining sea surface temperatures and changing current regimes during autumn and winter, may alter species distributions and migration patterns (Yamamoto et al., 2017; Harrison, Sunday & Rogers, 2019). Recorded water temperatures ranged from 23.6–25.7 °C in May 2021 to 23–28.3 °C in November 2024, reflecting subtropical thermal regimes and strong tidal mixing. Migratory responses by coastal fishes and elasmobranchs to changes in temperature, salinity, and prey availability are well documented (Sheaves, 2005; Nagelkerken et al., 2008). This study presented the first non-consecutive, inter-annual comparison of eDNA-derived fish communities, which serves as a baseline dataset for disentangling the complex environmental drivers shaping fish assemblages in the Urauchi River.

### Conservation threats in the urauchi river and considerations for edna-based in biodiversity monitoring

The Urauchi River, despite its well-known cultural and natural significance, is an ecologically sensitive and biodiversity-rich heritage area. This ecosystem is particularly fragile due to its limited spatial extent (∼1.5 km freshwater and ∼11 km brackish zones), which supports small and isolated fish populations vulnerable to environmental change (Suzuki & Mori, 2016b). Migratory and semi-marine species are especially at risk, as disruptions to ecological connectivity across habitats can interrupt life cycles and lead to local extirpations. Anthropogenic pressures further threaten this delicate system, which includes the following: construction of a large resort in 2004 linked to anaerobic sediment formation and goby declines (Okuda, 2005; Suzuki & Mori, 2016b); freshwater intake facility installed in 2015, which raised concerns about dry-season habitat degradation; and ongoing infrastructure developments such as bridge construction and fill mounds near the estuarine zone, which may alter tidal flow, increase sedimentation, and accelerate mangrove loss (Japan Tiger and Elephant Fund & Yamaneko Patrol, 2019). Such cumulative impacts highlight the urgency for long-term monitoring frameworks. Our study demonstrated that eDNA metabarcoding is well-suited to meet these needs, offering an effective means of detecting a wide spectrum of taxa.

Our study identified threatened species listed on the Japanese Red List (2020) and IUCN Red List (2025) (Fig. 2), including apex predators and nursery-dependent taxa. Although this eDNA survey did not detect all species recorded by long-term conventional sampling, it detected additional taxa despite being limited to only two sampling events. These results indicate that eDNA metabarcoding complements, and that increased sampling frequency would likely enhance detection (Valentini et al., 2016; Hallam et al., 2021; Sildever et al., 2023). The detection of abundant but taxonomically unresolved OTUs (Table S1) further points to current gaps in genetic reference databases.Taxonomic resolution may be constrained by marker-specific performance, PCR amplification bias, and high sequence similarity among closely related taxa, frequently limiting taxonomic assignment to the genus or family level (Kelly, Shelton & Gallego, 2019; Miya et al., 2015; Miya, 2022). Reliance on NCBI GenBank, which contains redundancy and occasional misidentifications, also required careful curation of sequence matches (Antinero et al., 2024; Wee et al., 2023; Valentini et al., 2016). Expanding regional barcode libraries, incorporating complementary markers, evaluating alternative bioinformatic pipelines, and refining bioinformatic processing workflows are therefore critical for improving taxonomic resolution and strengthening contamination control in eDNA-based biodiversity assessments (Antinero et al., 2024; Wee et al., 2023; Valentini et al., 2016).

Beyond taxonomic constraints, eDNA signals may reflect DNA transported from adjacent habitats influenced by hydrodynamics, seasonality, temperature, microbial activity, and sampling design (Barnes & Turner, 2016; Deiner & Altermatt, 2014; Harrison, Sunday & Rogers, 2019; Wee et al., 2023). For instance, unexpected detections of deepwater species within estuarine zones highlight the river’s strong marine–estuarine connectivity, which is likely mediated by tidal mixing, episodic surges, or predator-mediated eDNA transport. These findings emphasized the dynamic nature of riverine ecosystems and the need for fine-scale eDNA monitoring. Nevertheless, our study provides a molecular baseline for monitoring biodiversity in the Urauchi River and would contribute to broader conservation efforts within World Heritage landscapes. The success of global initiatives such as UNESCO’s eDNA Expeditions (Suominen et al., 2024), which employed citizen science to monitor threatened heritage sites, underscores the synergistic role of community stakeholders and molecular tools in safeguarding high-value ecosystems. As such, regular eDNA monitoring offers a promising early warning system for detecting population declines and community shifts while enabling proactive management. To strengthen future assessments, we recommend integrating eDNA-based monitoring surveys with targeted conventional surveys for validation, improving local reference databases, enhancing marker efficiency, conducting multi-depth and seasonal sampling, and incorporating hydrological modelling in the Urauchi River mangrove estuary. In addition, coupling species detection with high-resolution environmental data, trait-based analyses, and connectivity metrics (Sheaves, 2005) will support more robust species distribution models and inform targeted conservation planning.

## Conclusions

Our eDNA metabarcoding analysis detected remarkably high fish diversity in the UNESCO World Natural Heritage Site - Urauchi River. With the detection of unreported species in the previous checklist, the estimated total fish diversity in the Urauchi River may exceed what is currently known (∼407 species). Importantly, the detected species included endangered, cryptic, and deep-sea taxa, highlighting the ecological complexity and uniqueness of the river system. Spatial pattern with high species diversity observed at estuarine zones underscores the critical role of mangrove ecosystems as biodiversity hotspots and ecological corridors. This richness is observed across the freshwater–estuarine gradient and was significantly influenced by the environmental parameters such as salinity, temperature, and vegetation types. Therefore, our results established an important baseline for ecological monitoring, particularly on several detected threatened species. Despite its utility, eDNA metabarcoding has certain limitations, such as the absence of some taxa in the previous checklist and other historically recorded Red List taxa in the Urauchi River. It also relies on reference databases and is subject to detection uncertainties, as well as methodological and logistical constraints related to cost, infrastructure, and sampling design. This emphasised the need for improved sampling strategies, including multi-seasonal and depth-stratified approaches, to enhance the detection of low-abundance taxa. Integrating eDNA-based monitoring along with targeted conventional approaches may provide a more comprehensive assessment of fish assemblages. Amid increasing anthropogenic pressures and climate change, routine eDNA monitoring offers a practical early warning system for detecting ecological shifts and informing management. Global initiatives like UNESCO’s eDNA Expeditions, demonstrated eDNA metabarcoding as a sensitive and scalable approach to enhance conservation frameworks and ecosystem management. As the first molecular assessment of fish diversity conducted in this river system, this study provides an essential reference point for long-term biodiversity molecular monitoring to safeguard the ecological and cultural integrity of the Urauchi River.

## Supplemental Information

10.7717/peerj.21399/supp-1 Supplemental Information 1 Rarefaction curves depicting operational taxonomic unit (OTU) richness across individual samples (*n* = 42) Each curve represents the accumulation of observed OTUs as a function of raw sequencing depth (total number of reads per sample, obtained from the combined results of two enzymes, KAPA and Primestar). The x-axis indicates the number of reads, while the y-axis shows the corresponding number of OTUs detected. Sample labels are annotated at the terminal point of each curve, ordered by increasing sequencing depth. Curves were generated using the rarecure() function from the R package vegan, based on the OTU table extracted from the phyloseq object. r1, replicate 1; r2, replicate 2; two samples collected at each station.

10.7717/peerj.21399/supp-2 Supplemental Information 2 Heatmap showing the distribution and relative abundance of principal fish species detected in the Urauchi River Only species with a frequency of occurrence ≥45% across all samples (years, enzyme treatments, and sites) are displayed. Rows are ordered by total abundance. The color gradient reflects relative species abundance calculated using Hellinger-transformed read counts, with darker shades indicating higher abundance. Sampling site labels follow the format: Site-Year–Vegetation type. Vegetation types: SF, Subtropical Forest; SF-pm, Subtropical, Forest with patchy mangroves; MFsf, Mangrove Forest with subtropical forest; MF. Mangrove Forest; MFbf, Mangrove Forest with beach forest. This heatmap visualizes spatial and temporal patterns in fish community composition along the river’s freshwater–estuarine gradient.

10.7717/peerj.21399/supp-3 Supplemental Information 3 Cluster dendrogram of fish community composition in the Urauchi River, based on Bray–Curtis dissimilarity The analysis includes fish species with ≥30% frequency of occurrence across all samples. Labels indicate sampling sites, survey years (2021 or 2024), and location categories: RV, River; UP-e, Upper estuary; Mid-e, Middle estuary; LOW-e, Lower estuary. The dendrogram illustrates spatial and temporal clustering of fish assemblages along the freshwater–estuarine continuum, highlighting similarities and shifts in community structure across years and locations.

10.7717/peerj.21399/supp-4 Supplemental Information 4 Fish OTU table derived from eDNA analysis of the Urauchi River Feature ID: Unique sequence identifier; Sequence: Nucleotide string; Class, Order, Family, Genus: Taxonomic ranks were based from “All Japanese Fish Species Online, ver. 31 (Motomura, 2024)”; Status: (1) Species level - Sequence with ¿98.5% identity, forming monophyletic groups and matching confirmed species distributions, including (1*) Recognized as a distinct species in Japan, but pending formal scientific description (2) Genus level, (3) Family level, (4) Unannotated OTU- Low identity, but supported by relevant reads and occurrence (5) Deep-water species (0) garbage data - low identity or read counts, species with 1–2 base pair differences in the sequence had higher supporting reads, or not distributed in Japan; Memo: data- number of GenBank reference sequences submitted at analysis time; bpd- base pair differences; mono- monophyletic group; branching- minor intraspecies divergence; Previous Checklist: (✓) reported in Suzuki & Seno (2005), (-) not reported; Japan Red List: (CR, EN, VU, NT) conservation priority per Japan’s Ministry of the Environment (2020), (-) Least concern.

10.7717/peerj.21399/supp-5 Supplemental Information 5 Comparison of fish taxa listed in the checklist by Suzuki & Seno (2005) with the results from the present eDNA study ID No. and Checklist: Original identifier number and Japanesefish taxa listed in teh e previous checklist (Suzuki & Seno, 2005). Infraspecific taxon : (x) Treated as an infraspecific taxon - distinct in Suzuki & Seno (2005) but sharing the same scientific name (–) non-infraspecific taxon. Status: (1) Identified at the species level; (1*) Japanese-name taxa treated as distinct species but pending formal scientific description; (2) Identified at the genus level; (3) Identified at the family level. Species, Genus, Family: Taxonomic classification based on All Japanese Fish Species Online, ver. 31 (Motomura, 2024). eDNA detected (species, genus, family): (✓) indicates detection in the present eDNA study (-) not detected

10.7717/peerj.21399/supp-6 Supplemental Information 6 Photographic References List of complete references and attributions for images.

## Additional Information and Declarations

### Field Study Permissions

The following information was supplied relating to field study approvals (i.e., approving body and any reference numbers): Field permits were obtained from Okinawa District Forest Office, Forestry Agency, Japan with No. 2026 and No. 2085 (for 2021) and No. 6-1-24 (for 2024).

### Data Availability

The following information was supplied regarding data availability: The data and code are a vailable at OSF: Pananganan, B. G. S. (2026, April 8). Pananganan et. al. Retrieved from osf.io/p3wx7 The raw sequence data are available at NCBI BioProject: PRJNA1314965.
