# Supplementary figures and images for: eDNA reveals extraordinary fish diversity in the Urauchi River, Iriomote Island, Japan, a UNESCO World Heritage Site
Source: PeerJ. 2026 Jun 24;14:e21399. doi: 10.7717/peerj.21399 (PMC13310044; doi:10.7717/peerj.21399)

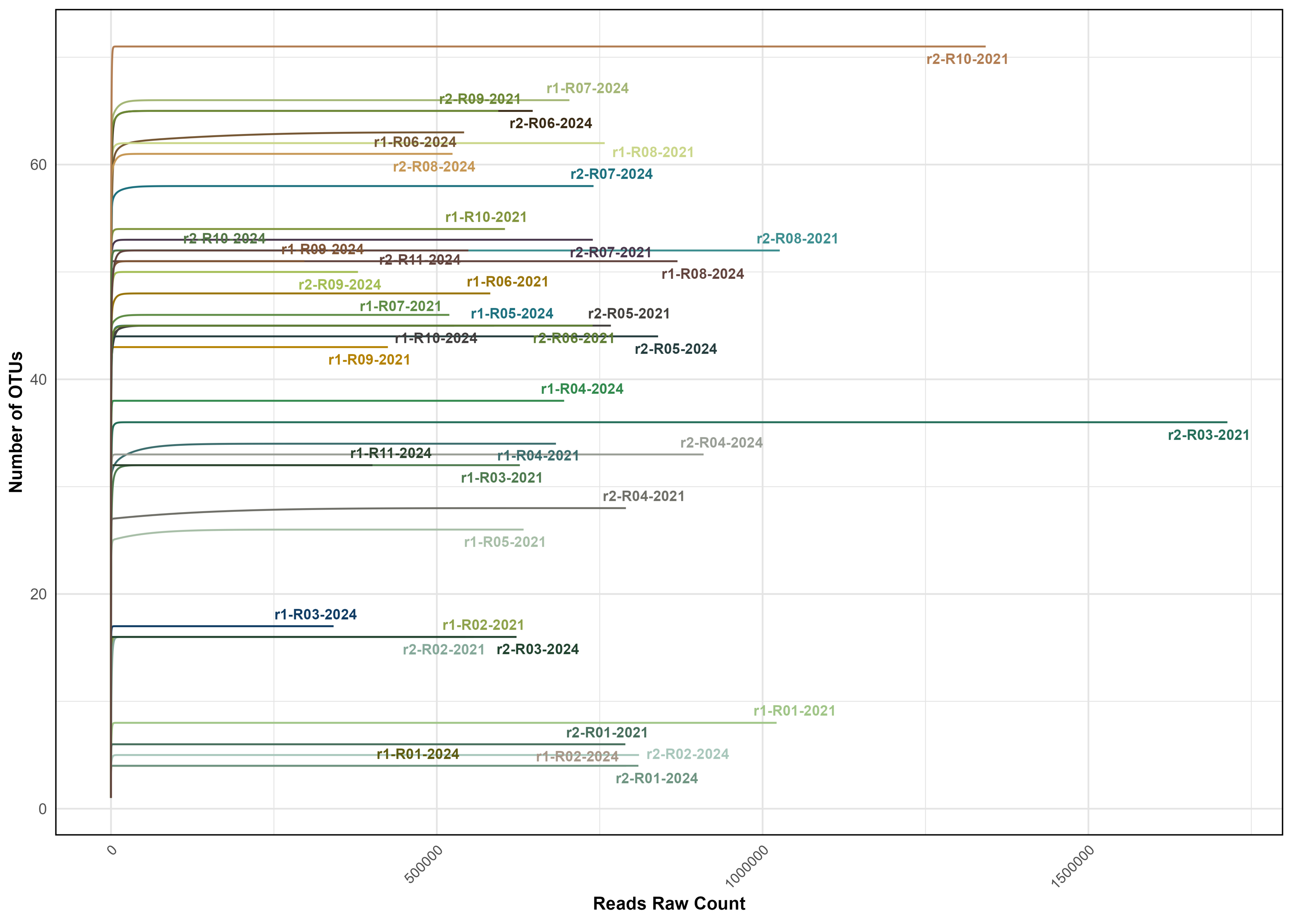

Supplement: Supplemental Information 1 — Each curve represents the accumulation of observed OTUs as a function of raw sequencing depth (total number of reads per sample, obtained from the combined results of two enzymes, KAPA and Primestar). The x-axis indicates the number of reads, while the y-axis shows the corresponding number of OTUs detected. Sample labels are annotated at the terminal point of each curve, ordered by increasing sequencing depth. Curves were generated using the rarecure() function from the R package vegan, based on the OTU table extracted from the phyloseq object. r1, replicate 1; r2, replicate 2; two samples collected at each station. [file peerj-14-21399-s001.png]

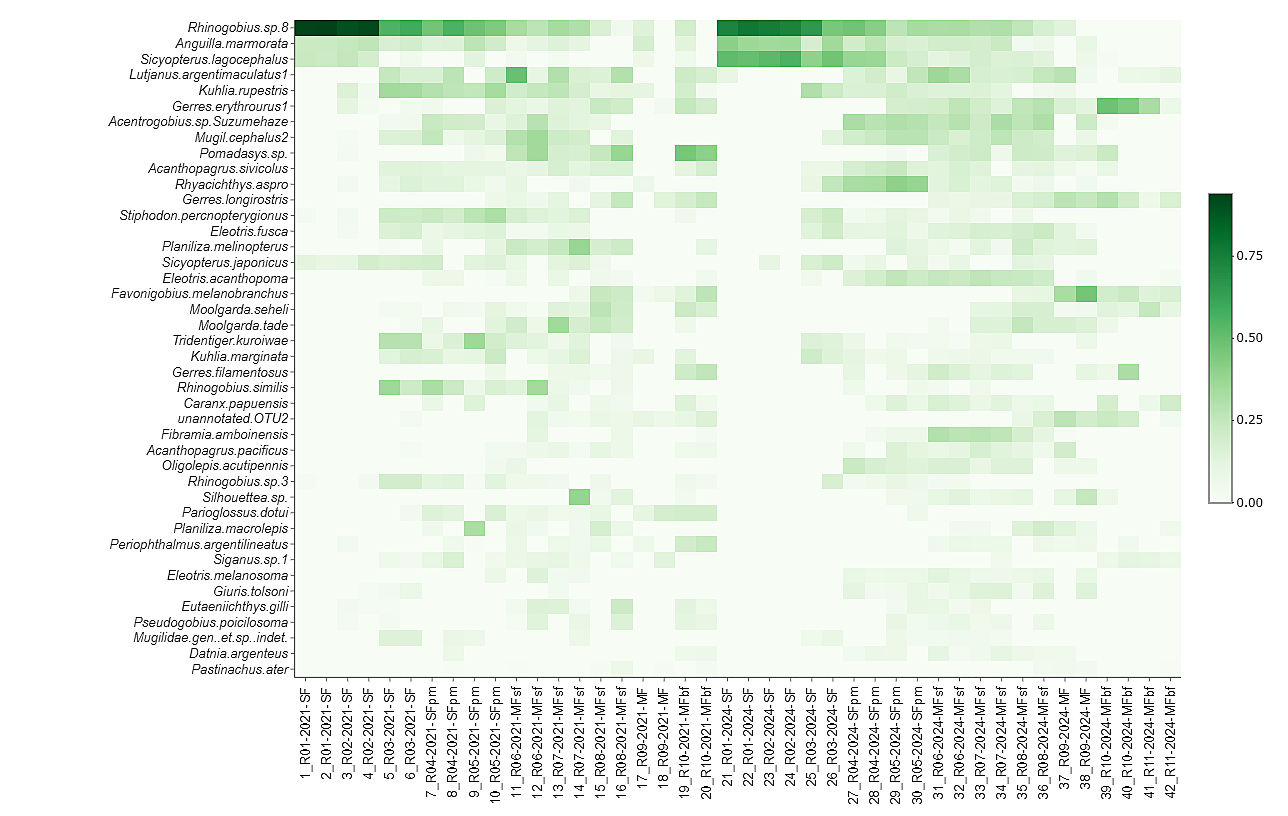

Supplement: Supplemental Information 2 — Only species with a frequency of occurrence ≥45% across all samples (years, enzyme treatments, and sites) are displayed. Rows are ordered by total abundance. The color gradient reflects relative species abundance calculated using Hellinger-transformed read counts, with darker shades indicating higher abundance. Sampling site labels follow the format: Site-Year–Vegetation type. Vegetation types: SF, Subtropical Forest; SF-pm, Subtropical, Forest with patchy mangroves; MFsf, Mangrove Forest with subtropical forest; MF. Mangrove Forest; MFbf, Mangrove Forest with beach forest. This heatmap visualizes spatial and temporal patterns in fish community composition along the river’s freshwater–estuarine gradient. [file peerj-14-21399-s002.png]

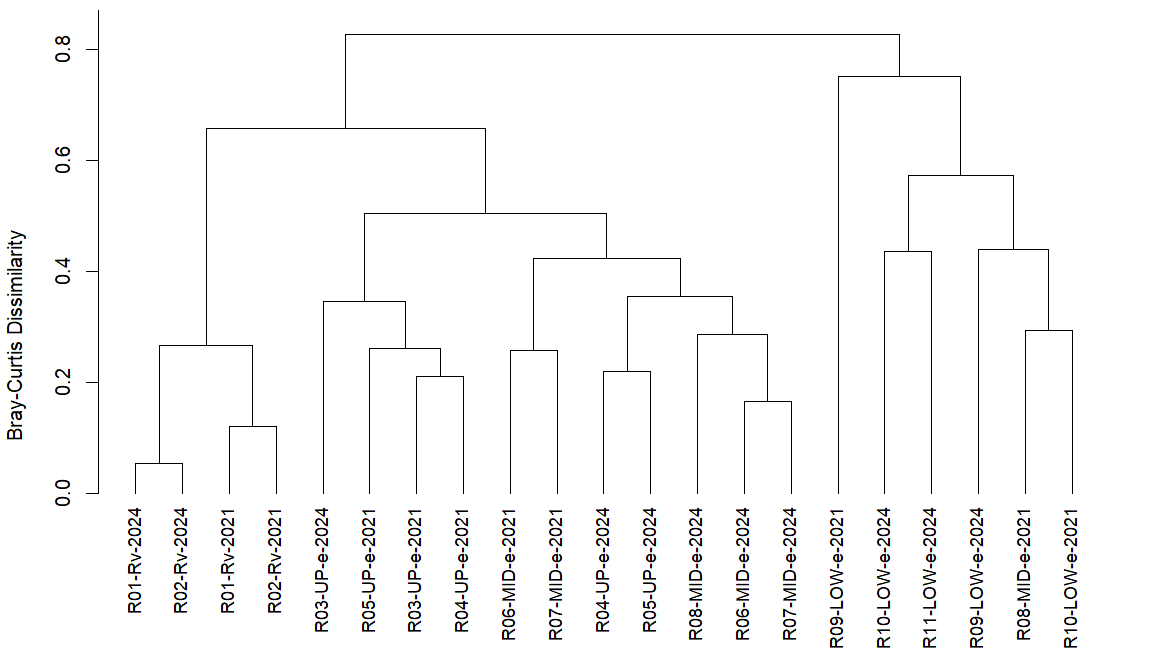

Supplement: Supplemental Information 3 — The analysis includes fish species with ≥30% frequency of occurrence across all samples. Labels indicate sampling sites, survey years (2021 or 2024), and location categories: RV, River; UP-e, Upper estuary; Mid-e, Middle estuary; LOW-e, Lower estuary. The dendrogram illustrates spatial and temporal clustering of fish assemblages along the freshwater–estuarine continuum, highlighting similarities and shifts in community structure across years and locations. [file peerj-14-21399-s003.png]
