# Supplementary material for: eDNA reveals extraordinary fish diversity in the Urauchi River, Iriomote Island, Japan, a UNESCO World Heritage Site
Source: PeerJ. 2026 Jun 24;14:e21399. doi: 10.7717/peerj.21399 (PMC13310044; doi:10.7717/peerj.21399)
Supplement: Supplemental Information 6 — List of complete references and attributions for images. [file peerj-14-21399-s006.docx]

## **Photographic References**

1. BIO Photography Group, Biodiversity Institute of Ontario. (2008, September 9). *Ophiocara porocephala = Ophiocara ophicephalus* [Photograph]. Australian National Fish Collection, CSIRO. <https://images.ala.org.au/image/6db40327-4350-478e-83cc-4c7f72c93963>
2. Bucol, A. (2014, April 22). *Caragobius urolepis (Bleeker, 1852)* [Photograph]. iNaturalist. <https://inaturalist.org/photos/30113855>. Licensed under CC BY-NC 4.0
3. Cassez, J.-P. (2021, April 4). *Pink Whipray (Pateobatis fai)* [Photograph]. iNaturalist. <https://www.inaturalist.org/photos/122379823>. Licensed under CC BY-NC 4.0
4. FAO. (1992). *FAO species catalogue. Vol. 14. Sillaginid fishes of the world (Family Sillaginidae): An annotated and illustrated catalogue of the Sillago, smelt or Indo-Pacific whiting species known to date* (FAO Fisheries Synopsis No. 125, Vol. 14, p. 87). Food and Agriculture Organization of the United Nations.
5. Hubert, N. (2016). *BIFD4519-16 – BOLD:AAP8873 (Planiliza melinoptera)* [Photograph]. Barcode of Life Data System.

<https://bins.boldsystems.org/index.php/Public_RecordView?processid=BIFD4519-16>. Licensed under CC BY 4.0

1. Luis, P. B. (2025, January 12). *Napoleon Wrasse (Cheilinus undulatus)* [Photograph]. iNaturalist. <https://www.inaturalist.org/photos/469525696>. Licensed under CC BY-NC 4.0
2. H.T. Cheng. (2016, May). (*Acanthopagrus sivicolus*) [Photograph]. iNaturalist. https://www.inaturalist.org/photos/3540373. Licensed under CC BY-NC 4.0.
3. National Fisheries Research and Development Institute. (2024). *BFPHL004-11 Lateral – BOLD:AAZ5305 (cf. Mesopristes cancellatus – Datnia cancellatus)* [Photograph]. Barcode of Life Data System. <https://bins.boldsystems.org/index.php/Public_RecordView?processid=BFPHL004-11>. Licensed under CC BY 4.0
4. Plume, S. (2024, May 18). *Broad Cowtail Stingray (Pastinachus ater)* [Photograph]. iNaturalist Australia. <https://www.inaturalist.org/photos/490487183>. Licensed under CC BY-NC 4.0
5. Randall, J. E. (n.d.). *Mesopristes argenteus = Datnia argenteus (Cuvier, 1829)* [Photograph]. Australian National Fish Collection, CSIRO. <https://images.ala.org.au/image/846388e0-29b9-4a1b-86aa-b7ec86dde63b>
6. Rosenstein, M. (2024, December 15). *Banded Archerfish (Toxotes jaculatrix)* [Photograph]. iNaturalist. <https://www.inaturalist.org/photos/459074897>. Licensed under CC BY-NC 4.0
7. Thompson, V. J., & Bray, D. J. (n.d.). *Hippichthys heptagonus* in Fishes of Australia. Museum Victoria. Retrieved July 24, 2025, from <https://fishesofaustralia.net.au/home/species/1532>
8. Senou, H. (1998, January 16). *Zenarchopterus dunckeri*, Iriomote-jima Island, Okinawa Prefecture, Japan [Photograph, KPM-NR 50640]. FishPix: Kanagawa Prefectural Museum of Natural History & National Museum of Nature and Science. <https://fishpix.kahaku.go.jp/fishimage-e/search.html>
9. Senou, H. (2010, June 17). *Sicyopterus lagocephalus* (male, immature), Chichi-jima Island, Ogasawara Islands, Tokyo Metropolis, Japan [Photograph, KPM-NR 49042]. FishPix: Kanagawa Prefectural Museum of Natural History & National Museum of Nature and Science. <https://fishpix.kahaku.go.jp/fishimage-e/search.html>
10. Senou, H. (2012, August 26). *Tylosurus crocodilus crocodilus*, west of Sagami Bay, Kanagawa Prefecture, Japan [Photograph, KPM-NR 106614]. FishPix: Kanagawa Prefectural Museum of Natural History & National Museum of Nature and Science. <https://fishpix.kahaku.go.jp/fishimage-e/search.html>
11. Senou, H. (2013, April 28). *Acanthopagrus pacificus*, Iriomote-jima Island, Okinawa Prefecture, Japan [Photograph, KPM-NR 107118]. FishPix: Kanagawa Prefectural Museum of Natural History & National Museum of Nature and Science. <https://fishpix.kahaku.go.jp/fishimage-e/search.html>
12. Senou, H. (2013, April 28). *Anguilla japonica* Temminck et Schlegel, 1847 [Photograph, KPM-NR 18995]. FishPix: Kanagawa Prefectural Museum of Natural History & National Museum of Nature and Science. <https://fishpix.kahaku.go.jp/fishimage-e/search.html>
13. Senou, H. (2015, July 25). *Tridentiger kuroiwae*, Amami-oshima Island, Kagoshima Prefecture, Japan (depth: 0.2 m) [Photograph, KPM-NR 108780]. FishPix: Kanagawa Prefectural Museum of Natural History & National Museum of Nature and Science. <https://fishpix.kahaku.go.jp/fishimage-e/search.html>
14. Senou, H. (2017, September 3). *Strongylura incisa*, Ie-jima Island, Okinawa Prefecture, Japan (depth: 0 m) [Photograph, KPM-NR 180624]. FishPix: Kanagawa Prefectural Museum of Natural History & National Museum of Nature and Science. <https://fishpix.kahaku.go.jp/fishimage-e/search.html>
15. Senou, H. (2018, August 5). *Ctenochaetus striatus*, Ishigaki-jima Island, Okinawa Prefecture, Japan [Photograph, KPM-NR 181444]. FishPix: Kanagawa Prefectural Museum of Natural History & National Museum of Nature and Science. <https://fishpix.kahaku.go.jp/fishimage-e/search.html>
16. Suzuki, T. (n.d.). *Acentrogobius viganensis =* ***Acentrogobius sp. (SUZUME HAZE)*** (Steindachner, 1893) [Photograph, KPM-NR 20101]. FishPix: Kanagawa Prefectural Museum of Natural History & National Museum of Nature and Science. <https://fishpix.kahaku.go.jp/fishimage-e/search.html>
17. Suzuki, T. (n.d.). *Bostrychus sinensis*, Iriomote-jima Island, Okinawa Prefecture, Japan [Photograph, KPM-NR 56437]. FishPix: Kanagawa Prefectural Museum of Natural History & National Museum of Nature and Science. <https://fishpix.kahaku.go.jp/fishimage-e/search.html>
18. Suzuki, T. (n.d.). *Chelon melinopterus* = **Planiliza melinopterus** (Valenciennes, 1836) [Photograph, KPM-NR 60280]. FishPix: Kanagawa Prefectural Museum of Natural History & National Museum of Nature and Science. <https://fishpix.kahaku.go.jp/fishimage-e/search.html>
19. Suzuki, T. (n.d.). *Favonigobius melanobranchus*, NW coast of Okinawa-jima Island, East China Sea, Okinawa Prefecture, Japan [Photograph, KPM-NR 45807]. FishPix: Kanagawa Prefectural Museum of Natural History & National Museum of Nature and Science. <https://fishpix.kahaku.go.jp/fishimage-e/search.html>
20. Suzuki, T. (n.d.). *Kuhlia rupestris*, Iriomote-jima Island, Yaeyama Islands, Okinawa Prefecture, Japan [Photograph, KPM-NR 131020]. FishPix: Kanagawa Prefectural Museum of Natural History & National Museum of Nature and Science. <https://fishpix.kahaku.go.jp/fishimage-e/search.html>
21. Suzuki, T. (n.d.). *Mugil cephalus*, Hyogo Prefecture, Japan [Photograph, KPM-NR 18000]. FishPix: Kanagawa Prefectural Museum of Natural History & National Museum of Nature and Science. <https://fishpix.kahaku.go.jp/fishimage-e/search.html>
22. Suzuki, T. (n.d.). *Parkraemeria saltator*, Iriomote-jima Island, Okinawa Prefecture, Japan [Photograph, KPM-NR 46777]. FishPix: Kanagawa Prefectural Museum of Natural History & National Museum of Nature and Science. <https://fishpix.kahaku.go.jp/fishimage-e/search.html>
23. Suzuki, T. (n.d.). *Rhyacichthys aspro*, Iriomote-jima Island, Okinawa Prefecture, Japan [Photograph, KPM-NR 55987]. FishPix: Kanagawa Prefectural Museum of Natural History & National Museum of Nature and Science. <https://fishpix.kahaku.go.jp/fishimage-e/search.html>
24. Suzuki, T. (n.d.). *Trypauchenopsis intermedia* Volz, 1903 [Photograph, KPM-NR 74304]. FishPix: Kanagawa Prefectural Museum of Natural History & National Museum of Nature and Science. <https://fishpix.kahaku.go.jp/fishimage-e/search.html>
25. Murase, A. (2016, February 9). *Spratelloides delicatulus*, Miyazaki Prefecture, Japan (depth: 26 m) [Photograph, KPM-NR 181939]. FishPix: Kanagawa Prefectural Museum of Natural History & National Museum of Nature and Science. <https://fishpix.kahaku.go.jp/fishimage-e/search.html>
26. Senou, H. (n.d.). *Eutaeniichthys gilli* Jordan et Snyder, 1901 [Gobiidae], Chiba Prefecture, Japan [Photograph, KPM-NR 57754 - 1]. FishPix: Kanagawa Prefectural Museum of Natural History & National Museum of Nature and Science. <https://fishpix.kahaku.go.jp/fishimage-e/search.html>
27. Senou, H. (n.d.). *Gerres longirostris*, Iriomote-jima Island, Okinawa Prefecture, Japan [Photograph, KPM-NR 59315]. FishPix: Kanagawa Prefectural Museum of Natural History & National Museum of Nature and Science. <https://fishpix.kahaku.go.jp/fishimage-e/search.html>
28. Senou, H. (2013, April 28). *Gerres erythrourus* (Bloch, 1791) [Gerreidae], Iriomote-jima Island, Yaeyama Islands, Ryukyu Islands, Okinawa Prefecture, Japan [Photograph, KPM-NR 107107 - 1, 2]. FishPix: Kanagawa Prefectural Museum of Natural History & National Museum of Nature and Science.
29. Senou, H. (n.d.). *Lutjanus argentimaculatus*, Yaku-shima Island, Kagoshima Prefecture, Japan [Photograph, KPM-NR 48037]. FishPix: Kanagawa Prefectural Museum of Natural History & National Museum of Nature and Science. <https://fishpix.kahaku.go.jp/fishimage-e/search.html>
30. Senou, H. (n.d.). Carcharhinus leucas (Valenciennes, 1839) [Carcharhinidae], Iriomote-jima Island, Yaeyama Islands, Ryukyu Islands, Okinawa Prefecture, Japan [Photograph, KPM-NR 55512 - 1]. FishPix: Kanagawa Prefectural Museum of Natural History & National Museum of Nature and Science. <https://fishpix.kahaku.go.jp/fishimage-e/search.html>
